# Diagnostic and prognostic role of nitroglycerin-induced dilation in patients with suspected vasospastic angina, combined with ergonovine provocation test

**DOI:** 10.1038/s41598-021-03338-0

**Published:** 2021-12-13

**Authors:** Minsu Kim, Albert Youngwoo Jang, Pyung Chun Oh, Soon Yong Suh, Kyounghoon Lee, Woong Chol Kang, Taehoon Ahn, Seung Hwan Han

**Affiliations:** 1 Division of Cardiology, Department of Internal Medicine, Gachon University College of Medicine, Gil Hospital, 21, Namdong-daero 774 beon-gil, Namdong-gu, Incheon, 21565 Republic of Korea; 2 Division of Cardiovascular Diseases, Department of Internal Medicine, Korea University, Anam Hospital, Seoul, Republic of Korea

**Keywords:** Cardiology, Cardiovascular diseases

## Abstract

The diagnostic and prognostic role of nitroglycerin-induced dilation (NID) combined with ergonovine provocation test in patients with suspected VSA patients is not clear. A total of 438 consecutive patients who underwent the ergonovine provocation test for the diagnosis of vasospastic angina (VSA) were enrolled. Patients with VSA (n = 52) had a significantly greater coronary response to ergonovine (− 84.3 ± 10.5% vs. − 38.4 ± 17.9%, *p* < 0.001) and NID (26.3 ± 31.0% vs. 12.5 ± 19.0%, *p* < 0.001) than non-VSA patients. However, positive NID (more than 13.8% dilation, n = 170) showed a poor accuracy (AUC 0.64 [95% CI: 0.56–0.73], *p* = 0.001, sensitivity 60.4%, specificity 61.3%) for the diagnosis of VSA by ergonovine provocation test. Major adverse cardiovascular events (MACE) occurred more frequently in the VSA group than in the non-VSA group (9.6% vs. 2.2%, *p* = 0.006). In addition, the positive NID group showed a lower rate of MACE than the negative NID group (1.2% vs. 4.3%, *p* = 0.021). Interestingly, the group of VSA with negative NID had poor prognosis than any other combinations (Log-rank, *p* < 0.0001). Although NID had a limited role in the detection of VSA defined by ergonovine provocation test, NID combined with the ergonovine provocation test has an additive prognostic role in the clinical outcomes in patients with suspected VSA.

## Introduction

Vasospastic angina (VSA) is a functional coronary vasomotor disorder that can lead to ischemic events, including sudden cardiac death, fatal arrhythmia, and acute coronary syndrome due to epicardial coronary artery spasm^1^. Ergonovine provocation test has shown high sensitivity (77–100%) and specificity (98–99%) for the detection of VSA and is known as a standard method for the diagnosis of VSA that cannot be diagnosed by non-invasive tests^2–4^. Nitroglycerin-induced dilation (NID) has been proposed as an important method for the detection of vasomotor function in a large-scale clinical trial (ABSORB II)^5^. However, the role of NID and combined with ergonovine provocation test in the diagnosis and prognosis in patients with suspected vasospastic angina is not well defined. Therefore, we tested the diagnostic and prognostic role of NID combined with the ergonovine provocation test in patients with suspected VSA patients.

## Results

### Patient characteristics

**Figure 1:**
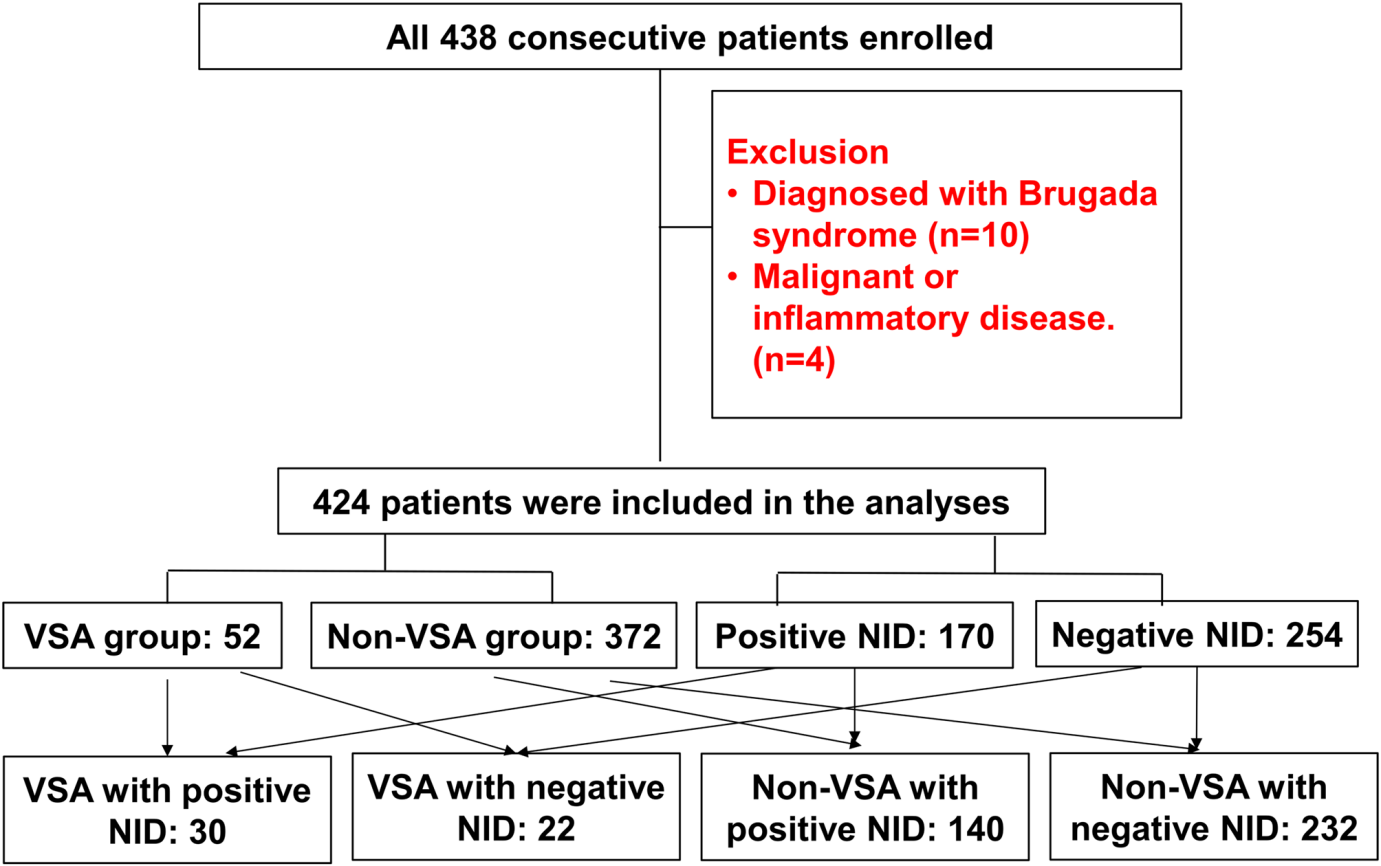
Study flow. Abbreviations: *VSA* vasospastic angina, *NID* nitroglycerin induced dilation.

A total of 438 consecutive patients who had suspicious symptoms for VSA and underwent CAG with the ergonovine provocation test (according to the clinician’s decision) were enrolled in the study. Fourteen patients were excluded as they did not meet the inclusion criteria. Finally, 424 patients (253 males, mean age 49.5 years) were included in this analysis. Of those patients, 52 (11.9%) were diagnosed with VSA by ergonovine provocation test and assigned to the VSA group; 254 patients (59.9%) showed negative NID and detailed groups of the combination according to the results of VSA and NID have shown in Fig. 1.

### Baseline clinical characteristics

Table 1 presents baseline clinical characteristics between the VSA and non-VSA groups by the ergonovine provocation test. The patients in the VSA group were older than those in the non-VSA group. The prevalence of hypertension and smoking was significantly higher in the VSA group than in the non-VSA group. Other baseline characteristics were similar between the two groups. The indications for the ergonovine provocation test did not differ significantly between the two groups. In both groups, the main indication for the ergonovine provocation test was chest pain while at rest (55.8% vs. 60.5%, respectively, *p* = 0.547). Baseline characteristics and indications for provocation tests between positive and negative NID groups are shown in Table 2. The frequency of atypical chest pain as an indication of provocation test was significantly lower in the positive NID group than in the negative NID group (7.1% vs. 15.0%, *p* = 0.014), but other indications did not differ significantly between the two groups.

**Table 1:** Baseline characteristics and indication for ergonovine provocation test between VSA and non-VSA groups.

	VSA (n = 52)	Non-VSA (n = 372)	*P* value
Age, years	52.7 $\pm$ 7.9	49.1 $\pm 13.5$	**0.006**
Male	37 (71.2)	216 (58.1)	0.072
BMI	25.3 $\pm 3.8$	24.6 $\pm 3.4$	0.233
HTN	22 (42.3)	100 (26.9)	**0.021**
Diabetes	3 (5.8)	24 (6.5)	0.850
Dyslipidemia	11 (21.2)	67 (18.0)	0.584
Current smoker	10 (19.2)	34 (9.1)	**0.047**
**Indication for an ergonovine provocation test**			
Resting pain	29 (55.8)	225 (60.5)	0.547
Exertional pain	5 (9.6)	13 (3.5)	0.056
Syncope	2 (3.8)	30 (8.1)	0.404
Arrest	12 (23.1)	50 (13.4)	0.091
Atypical chest pain	3 (5.8)	47 (12.6)	0.175
Palpitation	1 (1.9)	7 (1.9)	1.000

Data are expressed as number (percentages) or mean ± standard deviation.

Abbreviations: *VSA* vasospastic angina, *BMI* body mass index, *HTN* hypertension.

Significant values are in [bold].

**Table 2:** Baseline characteristics and indications for an ergonovine provocation test between positive and negative NID groups.

	Positive NID (n = 170)	Negative NID (n = 254)	*P* value
Age, years	50.6 $\pm$ 12.1	48.8 $\pm \;13.5$	0.221
Male	104 (61.2)	149 (58.7)	0.615
BMI	24.7 $\pm \;3.2$	24.8 $\pm \;3.6$	0.702
HTN	48 (28.2)	74 (29.1)	0.913
Diabetes	10 (5.9)	17 (6.7)	0.840
Dyslipidemia	27 (15.9)	51 (20.1)	0.307
Current smoker	23 (13.5)	21 (8.3)	0.104
**Indication for an ergonovine provocation test**			
Resting pain	103 (60.6)	151 (59.4)	0.840
Exertional pain	11 (6.5)	7 (2.8)	0.084
Syncope	17 (10.0)	15 (5.9)	0.135
Arrest	24 (14.1)	38 (15.0)	0.889
Atypical chest pain	12 (7.1)	38 (15.0)	**0.014**
Palpitation	3 (1.8)	5 (2.0)	1.000

Data are expressed as number (percentages) or mean ± standard deviation.

Abbreviations: *NID* nitroglycerin induced dilation, *BMI* body mass index, *HTN* hypertension.

Significant values are in [bold].

### Correlation between the coronary response to ergonovine and nitroglycerin

**Figure 2:**
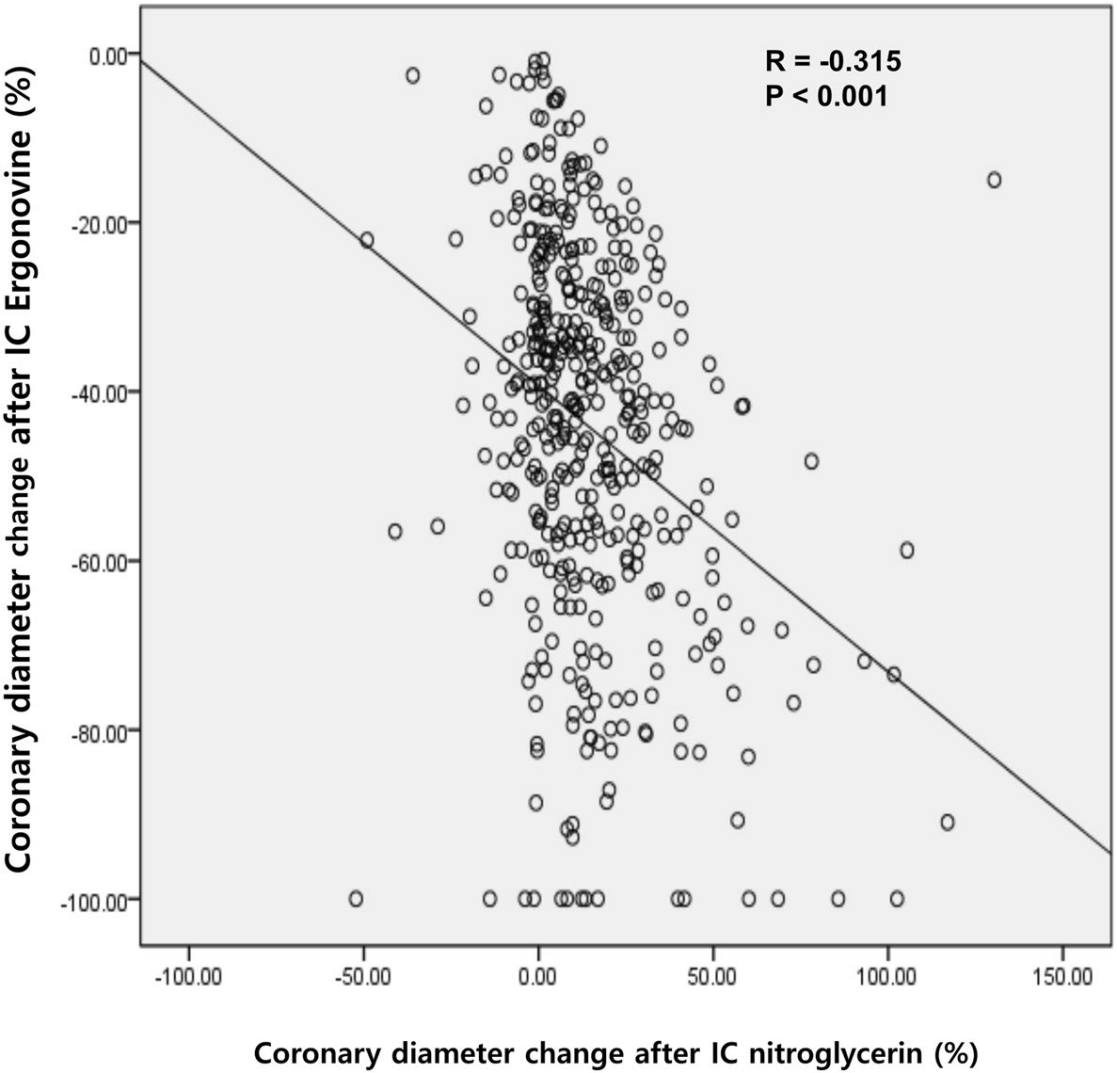
Correlation between coronary response to ergonovine and nitroglycerin. Abbreviations: *IC* intracoronary.

**Figure 3:**
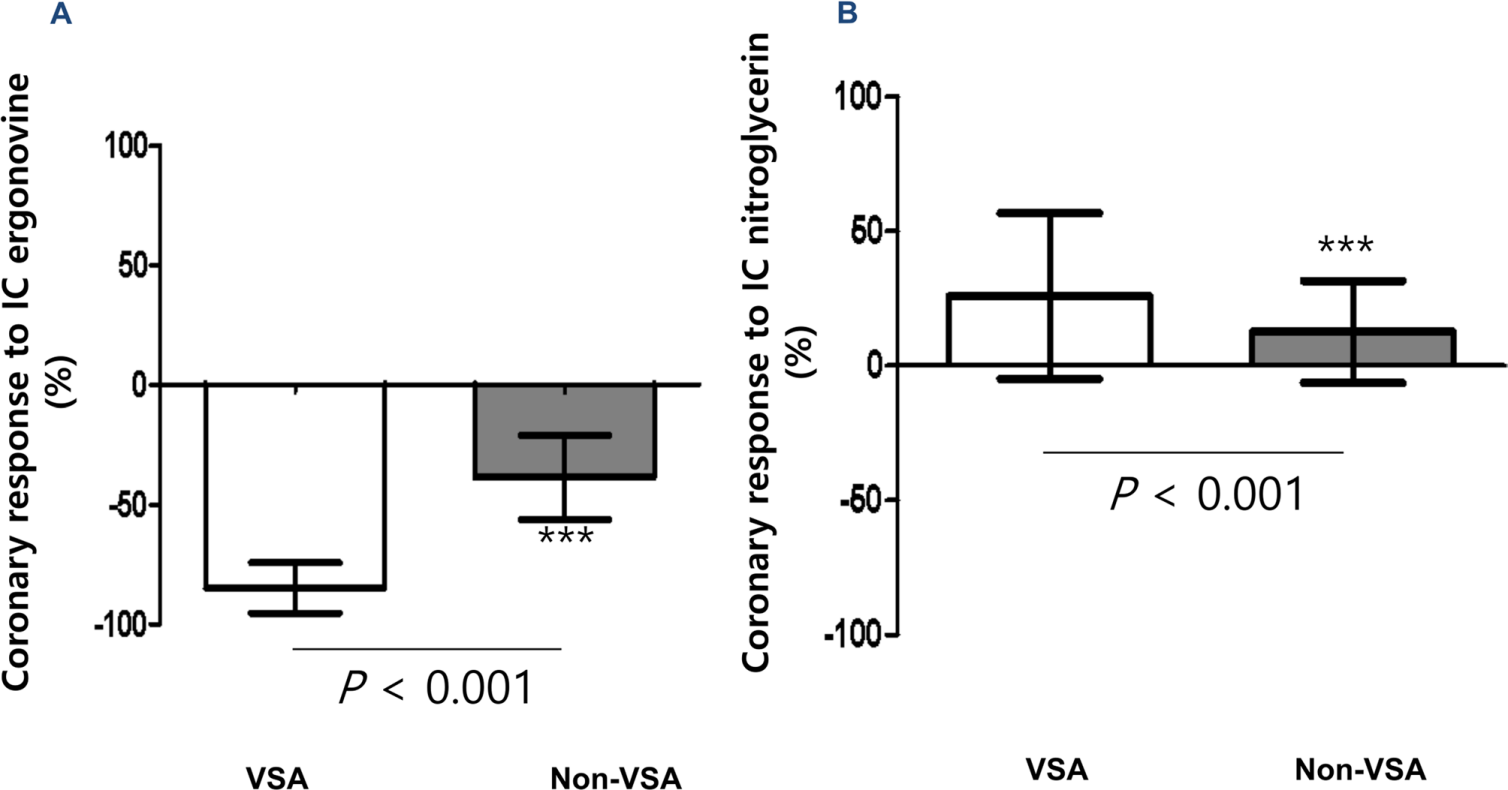
Comparison of coronary response to ergonovine and nitroglycerin between VSA and non-VSA groups. Bars indicate mean, and lines indicate standard deviation of the mean. For comparison of VSA with Non-VSA; *p* < 0.05*, *p* < 0.01**, *p* < 0.001***. Abbreviations: *IC* intracoronary, *VSA* vasospastic angina.

Coronary response to ergonovine showed a significant weak inverse correlation with NID (*r* = − 0.315, *p* < 0.001, Fig. 2). Patients with VSA had a significantly greater coronary response to ergonovine (− 84.7 ± 10.6% vs. − 38.4 ± 17.7%, *p* < 0.001) and NID (25.9 ± 30.8% vs. 12.6 ± 19.0%, *p* < 0.001) than non-VSA patients (Fig. 3A, B).

### Prediction of NID for the diagnosis of VSA by ergonovine provocation test

**Figure 4:**
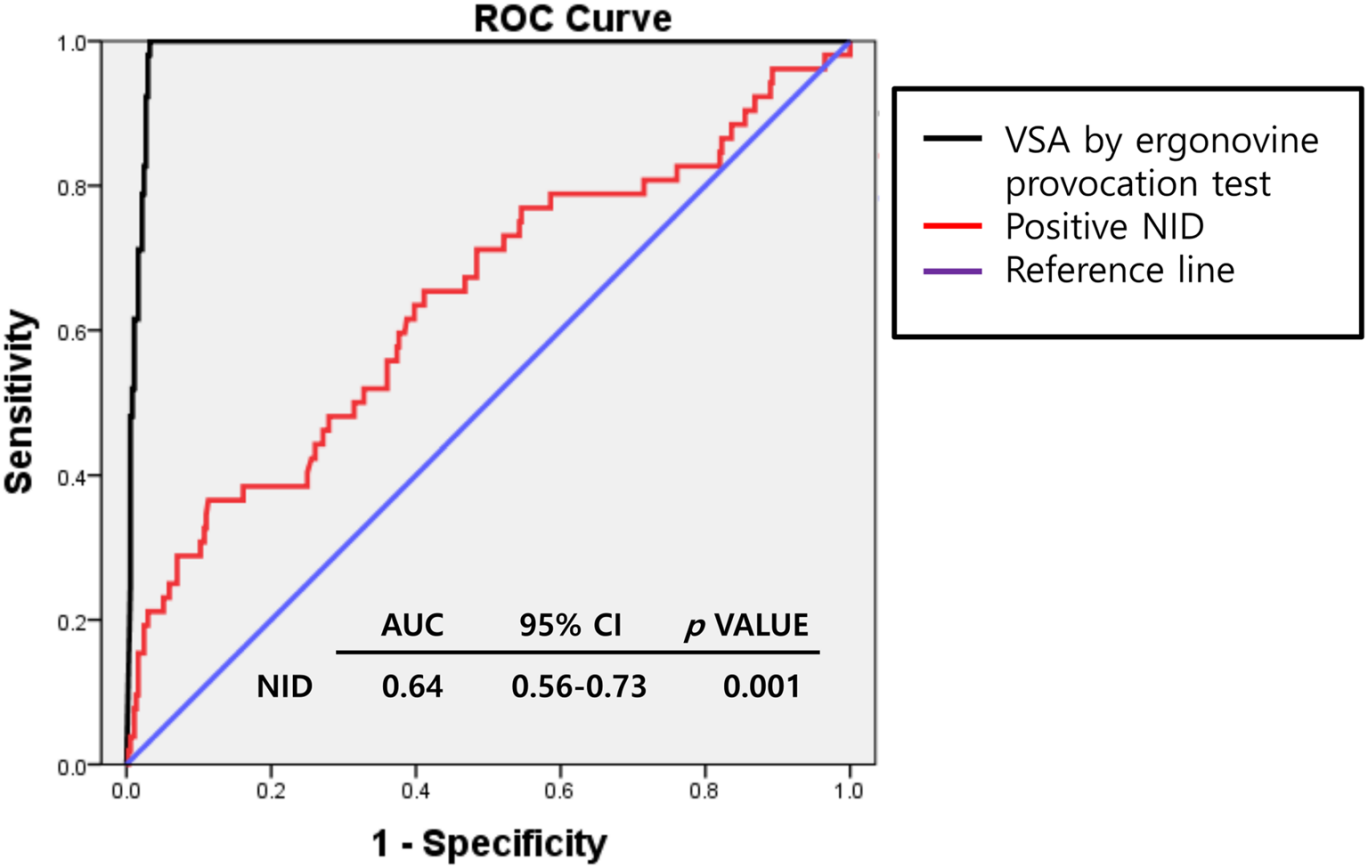
Receiver operating characteristic (ROC) curve analysis of the coronary response of IC nitroglycerin infusion for positive VSA patients according to ergonovine provocation test. Abbreviations: *ROC* receiver operating characteristic, *AUC* area under the curve, *CI* confidence interval, *VSA* vasospastic angina, *NID* nitroglycerin-induced dilation.

According to ROC curve analysis, the ideal cut-off value of NID for the diagnosis of VSA by ergonovine provocation test was more than 13.8% of diameter increase after IC nitroglycerin infusion. The prediction of positive NID (defined as more than 13.8% vasodilation by IC nitroglycerin) showed poor accuracy (AUC 0.64 [95% CI: 0.56–0.73], sensitivity 60.4%, specificity 61.3%, positive predictive value 17.6%, negative predictive value 91.3%) for the diagnosis of VSA by the ergonovine provocation test (Fig. 4).

### Medical treatments in the follow-up period

For the treatment of VSA, CCBs were prescribed to all patients (100%) of the VSA group, and long-acting nitrates were the second most frequently prescribed drugs (80.8%). In addition, antiplatelet agents, statins, angiotensin-converting enzyme inhibitors, angiotensin type II receptor blockers, and beta-blockers were used in 15 (28.8%), 25 (48.1%), 3 (5.8%), 7 (13.5%), and 5 patients (9.6%), respectively. The use of combination drug therapy with CCBs and nitroglycerin was significantly more frequent in the VSA group than in the non-VSA group (80.8% vs 34.9%, *p* < 0.0001), and the prescription of statin was less common in the non-VSA group (48.1% vs. 31.5%, *p* = 0.027) (Table 3). The use of beta-blockers was rare, but the frequency was similar in the VSA and non-VSA groups (9.6% vs. 13.7%, *p* = 0.516).

**Table 3:** Medical treatment during the follow-up period.

	VSA (n = 52)	Non-VSA (n = 372)	*P* value
Calcium channel blockers	52 (100)	249 (66.9)	**< 0.0001**
Long-acting nitrates	42 (80.8)	151 (40.6)	**< 0.0001**
Antiplatelet agents	15 (28.8)	98 (26.4)	0.739
Statins	25 (48.1)	117 (31.5)	**0.027**
ACE-Is	3 (5.8)	15 (4.0)	0.473
ARBs	7 (13.5)	59 (15.9)	0.838
Beta-blockers	5 (9.6)	51 (13.7)	0.516
Combination drug therapy	42 (80.8)	130 (34.9)	**< 0.0001**

Data are expressed as numbers (percentages).

Abbreviations: *VSA* vasospastic angina, *ACE-Is* angiotensin-converting enzyme inhibitors, *ARBs* angiotensin type II receptor blockers.

Significant values are in [bold].

### Comparison of clinical outcomes between VSA and non-VSA groups by ergonovine provocation test

During the median follow-up period of 17.3 (interquartile range [IQR], 6.2–33.2) months, MACE occurred more frequently in the VSA group than in the non-VSA group (9.6% vs. 2.2%, Log-Rank *p* = 0.006) even with more intensive medical treatments (Table 4).

**Table 4:** Comparison of MACE in VSA and non-VSA groups by ergonovine provocation test.

	VSA (n = 52)	Non-VSA (n = 372)	Log-rank *p* value
MACE	5 (9.6)	8 (2.2)	**0.006**
Cardiac death	3 (5.8)	0 (0)	**< 0.0001**
ACS	1 (1.9)	0 (0)	**0.008**
Fatal arrhythmia	0 (0)	7 (1.9)	0.317
Syncope	1 (1.9)	1 (0.3)	0.113
Hard MACE (cardiac death, ACS, fatal arrhythmia)	4 (7.7)	7( 1.9)	**0.021**
Re-hospitalization or ER visit for angina attack	6 (11.5)	32 (8.6)	0.390

Data are expressed as numbers (percentages).

Abbreviations: *MACE* major adverse cardiovascular event, *VSA* vasospastic angina, *ACS* acute coronary syndrome, *ER* emergency room.

Significant values are in [bold].

**Figure 5:**
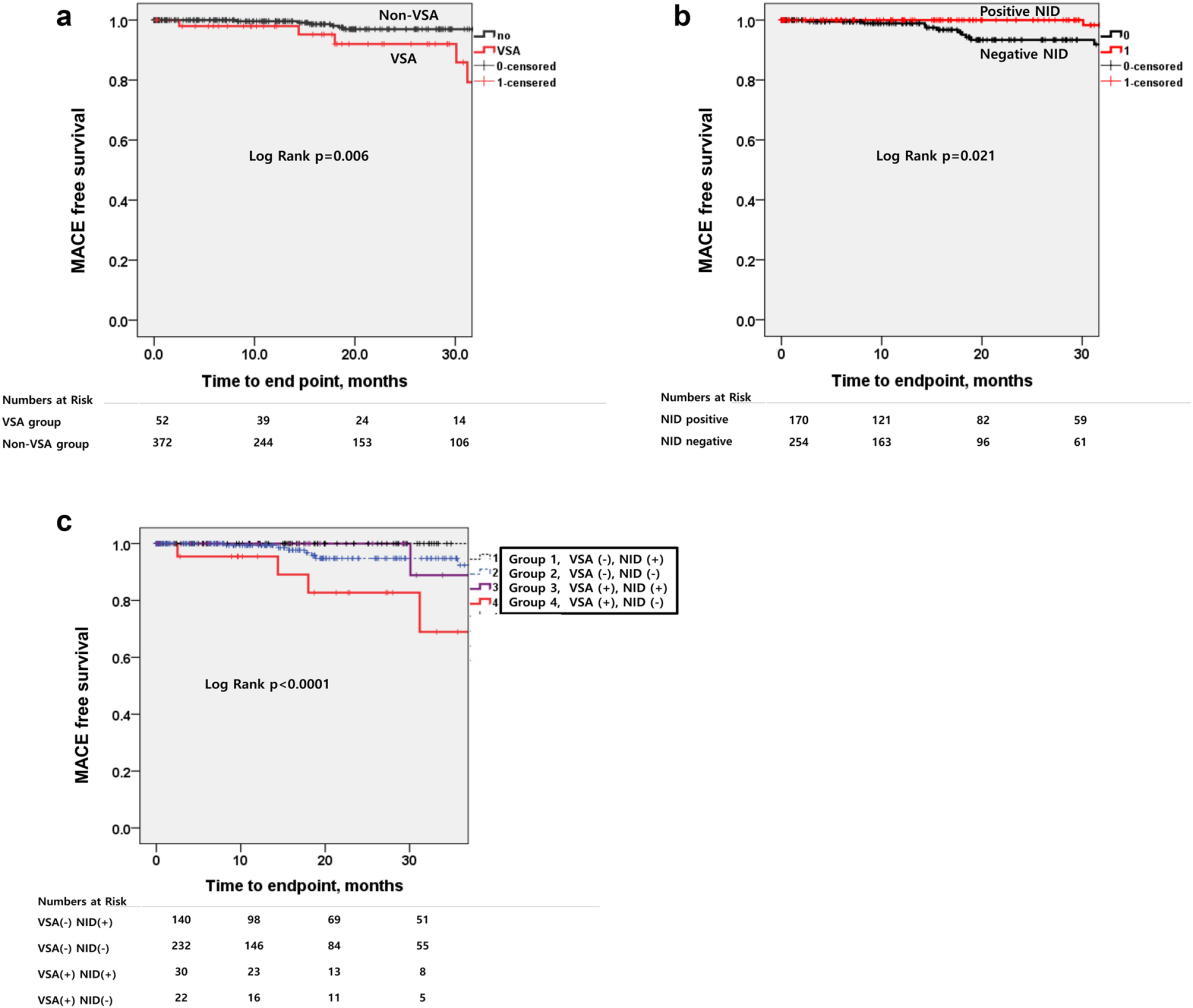
Kaplan–Meier survival curve analysis for the follow-up periods. (**a**) Kaplan–Meier survival curve in patients grouped according to the results of ergonovine provocation test. (**b**) Kaplan–Meier survival curve in patients grouped according to the results of NID. (**c**) Kaplan–Meier survival curve in patients grouped according to the results of ergonovine provocation test and NID. Abbreviations: *MACE* major adverse clinical events, *VSA* vasospastic angina, *NID* nitroglycerin induced vasodilation.

Figure 5A shows the Kaplan–Meier survival curve for the primary endpoint. MACE-free survival was significantly lower in the VSA group than in the non-VSA group. Of interest, the rate of cardiac death was significantly higher in the VSA group than in the non-VSA group (5.8% vs. 0%, Log-Rank *p* < 0.0001). However, with respect to re-hospitalization or ER visits for angina attack as the secondary endpoint, there was no significant difference between the two groups (11.5% vs. 8.6%, Log-Rank *p* = 0.390, Table 4).

### Comparison of clinical outcomes between positive NID and negative NID groups

MACE occurred less often in the positive NID group (1.2% vs. 4.3%, Log-Rank *p* = 0.021) (Table 5). Figure 5b shows the corresponding Kaplan–Meier curves for the primary endpoint. MACE-free survival was significantly lower in the negative NID group. Re-hospitalization or ER visits for angina attacks were also more frequent in the negative NID group (positive NID group 8.2% vs. negative NID group 9.4%, Log-Rank *p* = 0.034, Table 5).

**Table 5:** Comparison of MACE in positive NID and negative NID groups.

	Positive NID (n = 170)	Negative NID (n = 254)	Log-rank *p* value
MACE	2 (1.2)	11 (4.3)	**0.021**
Cardiac death	0 (0)	3 (1.2)	0.132
ACS	1 (0.6)	0 (0)	0.309
Fatal arrhythmia	1 (0.6)	6 (2.4)	0.065
Syncope	0 (0)	2 (0.8)	0.192
Hard MACE (cardiac death, ACS, fatal arrhythmia)	2 (1.2)	9 (3.5)	0.052
Rehospitalization or ER visit for angina attack	14 (8.2)	24 (9.4)	**0.034**

Data are expressed as numbers (percentages).

Abbreviations: *MACE* major adverse cardiovascular event, *NID* nitroglycerin induced dilation, *ACS* acute coronary syndrome, *ER* emergency room.

Significant values are in [bold].

### Baseline characteristics between 4 groups derived by the combinations of the results of ergonovine provocation and NID test

Table 6 shows the prevalence of current smoking was significantly different among 4 groups (*p* = 0.047) and was higher in the patients with VSA group (Group 3 or Group 4) than in the non-VSA group regardless of NID results. Regarding the indications for the ergonovine provocation test, exertional chest pain was more common in VSA with positive NID group (group 3) and cardiac arrest was more common in VSA with negative NID group (group 4). Other baseline clinical characteristics and laboratory data did not differ between the 4 groups(Table 6).

**Table 6:** Baseline characteristics and indications for an ergonovine provocation test between 4 groups derived from the combinations of VSA and NID results.

	Group1 VSA(−)/NID(+) (n = 140)	Group2 VSA(−)/NID(−) (n = 232)	Group3 VSA(+)/NID(+) (n = 30)	Group4 VSA(+)/NID(−) (n = 22)	*P* value
Age, years	50.1 $\pm \;12.8$	48.4 $\pm \;13.9$	52.9 $\pm \;7.8$	52.3 $\pm \;8.3$	0.171
Male	82(58.6)	134(57.8)	22(73.3)	15(68.2)	0.336
BMI, kg/m^2^	24.5 $\pm \;3.0$	24.8 $\pm \;3.6$	25.4 $\pm \;3.7$	25.1 $\pm \;4.1$	0.583
HTN	35(25.0)	65(28.0)	13(43.3)	9(40.9)	0.126
Diabetes	9(6.4)	15(6.5)	1(3.3)	2(9.1)	0.889
Dyslipidemia	20(14.3)	47(20.3)	7(23.3)	4(18.2)	0.433
Current smoker	17(12.1)	17(7.4)	6(20.0)	4(18.2)	**0.047**
**Indication for an ergonovine provocation test**					
Resting pain	83(59.3)	142(61.2)	20(66.7)	9(40.9)	0.255
Exertional pain	7(5.0)	6(2.6)	4(13.3)	1(4.5)	**0.049**
Syncope	15(10.7)	15(6.5)	2(6.7)	0(0)	0.285
Arrest	21(15.0)	29(12.5)	3(10.0)	9(40.9)	**0.011**
Atypical chest pain	11(7.9)	36(15.5)	1(3.3)	2(9.1)	0.065
Palpitation	3(2.1)	4(1.7)	0(0)	1(4.5)	0.615
**laboratory data**					
Hemoglobin, g/dL	13.4 $\pm$ 1.5	13.8 $\pm$ 1.7	13.8 $\pm$ 1.3	13.6 $\pm$ 1.5	0.354
FBS, mg/dL	98.18 $\pm$ 20.5	$93.74\; \pm$ 14.4	97.8 $\pm$ 11.3	95.5 $\pm$ 14.2	0.889
HbA1c, %	5.6 $\pm \;0.6$	5.8 $\pm \;2.8$	5.5 $\pm \;0.5$	5.5 $\pm \;0.4$	0.233
Total-C, mg/dL	165.6 $\pm \;35.5$	167.3 $\pm \;34.3$	165.9 $\pm \;30.8$	156.8 $\pm \;33.8$	0.684
Triglyceride, mg/dL	132.7 $\pm \;70.0$	134.7 $\pm \;88.2$	159.9 $\pm \;83.5$	135.9 $\pm \;81.7$	0.505
HDL-C, mg/dL	43.7 $\pm \;11.8$	45.6 $\pm \;12.8$	40.38 $\pm \;9.1$	40.7 $\pm \;9.0$	0.090
LDL-C, mg/dL	96.1 $\pm \;31.0$	97.6 $\pm \;29.2$	99.6 $\pm \;28.6$	116.9 $\pm \;98.2$	0.161
hsCRP, mg/dL	0.3 $\pm \;0.5$	0.3 $\pm \;0.7$	0.4 $\pm \;0.7$	0.5 $\pm \;0.9$	0.444
Creatinine, mg/dL	0.7 $\pm \;0.1$	0.8 $\pm \;0.6$	0.6 $\pm \;0.2$	0.8 $\pm \;0.5$	0.641

Data are expressed as numbers (percentages) or mean ± standard deviation.

Abbreviations: *VSA* vasospastic angina, *NID* nitroglycerin induced vasodilation, *BMI* body mass index, *HTN* hypertension, *FBS* fasting blood sugar, *total-C* total cholesterol, *HDL-C* HDL cholesterol, *LDL-C* LDL cholesterol, *hsCRP* high sensitivity C reactive protein.

Significant values are in [bold].

### Prognostic role of NID combined with ergonovine provocation test

The MACE-free survival was significantly lower in the group of VSA with negative NID (group 4) than in any other combinations (Log-Rank *p* < 0.0001, Table 7, Fig. 5c). In addition, the incidences of cardiac death, syncope, hard MACE, and rehospitalization or ER visit for angina attack were greater in the group of VSA with negative NID (group 4) than in any other combinations (Log-Rank *p* < 0.0001, *p* = 0.044, *p* = 0.006, *p* = 0.017, respectively, Table 7).

**Table 7:** Clinical outcomes between 4 groups derived from the combination of VSA and NID results.

	Group1 VSA(−)/NID(+) (n = 140)	Group2 VSA(−)/NID(−) (n = 232)	Group3 VSA(+)/NID(+) (n = 30)	Group4 VSA(+)/NID(−) (n = 22)	*P* value
MACE	1 (0.7)	7 (3.0)	1 (3.3)	4 (18.2)	**< 0.0001**
Cardiac death	0	0	0	3 (13.6)	**< 0.0001**
ACS	0	0	1 (3.3)	0	**0.006**
Fatal arrhythmia	1 (0.7)	6 (2.6)	0	0	0.209
Syncope	0	1 (0.4)	0	1 (4.5)	**0.044**
Hard MACE (cardiac death, ACS, fatal arrhythmia)	1 (0.7)	6 (2.6)	1 (3.3)	3 (13.6)	**0.006**
Rehospitalization or ER visit for angina attack	13 (9.3)	19 (8.2)	1 (3.3)	5 (22.7)	**0.017**

Data are expressed as numbers (percentages).

Abbreviations: *VSA* vasospastic angina, *NID* nitroglycerin induced dilation, *MACE* major adverse cardiovascular event, *ACS* acute coronary syndrome, *ER* emergency room.

Significant values are in [bold].

## Discussion

To our knowledge, this study is the first study to investigate the diagnostic and prognostic role of NID combined with the ergonovine provocation test in the diagnosis and prognosis in patients with suspected vasospastic angina.

The salient findings from this study of suspected VSA patients are (1) NID showed a significant but weak inverse correlation with coronary response to ergonovine; however, (2) a positive response of NID (more than 13.8% vasodilation after intracoronary nitroglycerin injection) cannot effectively predict the result of ergonovine provocation test (the standard provocation test for the diagnosis of VSA); (3) although NID did not predict the results of the ergonovine provocation test, NID itself have a prognostic role in patients with suspected VSA; and (4) interestingly, group of VSA with negative NID showed the worst prognosis of any other groups (Log-Rank *p* < 0.0001). Therefore, although NID had a limited role in the detection of VSA defined by ergonovine provocation test, it did provide an additive prognostic role in the clinical outcomes in suspected VSA patients.

Although several alternative diagnostic options for VSA have been introduced, the angiographic provocation test with ergonovine is known as the standard provocation test method for the detection of VSA if VSA is not diagnosed by non-invasive tests^2^. The coronary artery spasm provocation test is recommended by the European Society of Cardiology (Class IIa, level of evidence B) and Japanese Circulation Society (Class I) in patients with suspected VSA^6,7^. Ergonovine, in patients with VSA, provokes coronary hyper-constriction by the activation of serotonergic receptors with a subsequent increase in calcium influx in vascular smooth muscle cells^8^.

Although the ergonovine provocation test is known as a standard method for the detection of VSA, in real clinical practice, the rate of performing the ergonovine provocation test is relatively low, because it is time-consuming and presents certain risks. In this regard, some researchers have introduced simpler methods for the diagnosis of vasospastic angina. In one retrospective study, the predictive value of NID for a positive acetylcholine provocation test was evaluated^9^. The authors of this study concluded that NID is a possible predictor for a positive result of the acetylcholine provocation test. However, this study was limited to the evaluation of the predictive value of NID, and the investigation was limited to the RCA. Moreover, the prognostic role of NID was not reported. In another study, an alternative method was suggested, one that uses a double-acquisition coronary CT angiography protocol in the presence and absence of intravenous nitroglycerin for detecting VSA^10^. The coronary CT angiography results in this study had relatively high sensitivity (73%) and specificity (100%) for predicting positive results of the ergonovine provocation test. However, the small number of enrolled patients (N = 20) limited the researchers’ ability to identify predictive value. Given the limitations of these published studies, our current work gives some important insights into the role of NID in the detection of VSA in real clinical practice. Because there is no previous study investigating the criteria of NID in coronary arteries, in the current study, we sought the criteria of NID which predict the result of the ergonovine provocation test. According to ROC curve analysis, the ideal cut-off value of NID for the diagnosis of VSA by ergonovine provocation test was more than 13.8% of diameter increase after IC nitroglycerin infusion (positive NID). However, prediction of positive NID (define by more than 13.8% vasodilation by IC nitroglycerin) had low accuracy (AUC 0.64 [95% CI: 0.56–0.73], sensitivity 60.4%, specificity 61.3%, positive predictive value 17.6%, negative predictive value 91.3%) for the diagnosis of VSA by the ergonovine provocation test. Therefore, our results demonstrate that the NID test cannot replace the ergonovine provocation test in the diagnosis of VSA.

Compared with the previous studies^9,10^, our study has several advantages. First, our analysis was conducted with a relatively large number of patients who were enrolled consecutively. Second, we evaluated the prognostic value of NID combined with the ergonovine provocation test. In addition, we used an automated quantitative coronary analysis program for a more precise measure of angiographic data.

Previously, NID was used to assess endothelial independent function and regarded as a control test for an endothelium-dependent function test. Endothelial function plays an important role in the pathogenesis of atherosclerosis and predicts cardiovascular outcomes, especially in patients with early coronary atherosclerosis^11,12^. Recently, the diagnostic and prognostic role of NID in detecting patients with high-risk cardiovascular disease was described by some investigators^13,14^. Impairment of NID is initiated at a later stage of atherosclerosis and can be used as a marker of atherosclerosis and risk stratification of high-risk patients with cardiovascular disease^15,16^. In one study, the combination of endothelial function test (flow-mediated dilation, FMD) and NID measurements could more accurately predict cardiovascular events than FMD alone^15^. Given these findings, our current results demonstrate NID in the coronary artery also plays an important role in the prognosis of suspected VSA patients. In addition, our results reinforce the finding that ergonovine provocation test in patients with suspected VSA plays an important role in the prediction of MACE^13,14,17,18^. Of interest, the combined results of ergonovine provocation test and NID provide important information on the prognosis of suspected VSA patients.

## Limitations

This study has several limitations. First, although the patients in this study were prospectively enrolled in the VSA registry which evaluates the risk factors and clinical outcomes in patients with suspected VSA who underwent ergonovine provocation test, the current analysis was a retrospective, cross-sectional study. Second, transient coronary artery occlusion (> 70% luminal diameter narrowing) as a cut-off value for the diagnosis of coronary artery spasm. Definitive VSA could be diagnosed if transient total or subtotal coronary artery occlusion (≥ 90% constriction) with angina and ischemic ECG changes occurred in response to the provocative stimulus. However, we quote our criterion to avoid any risk for the patients because a higher dose of ergonovine injection would be required to induce subtotal or total obstruction. Third, although all patients diagnosed with VSA by the ergonovine provocation test received antianginal medication, the prescription type and duration for each patient were at the physician’s discretion.

## Conclusions

Although we found that NID had a limited role in the detection of VSA defined by the ergonovine provocation test, NID, especially combined with ergonovine provocation test, does provide an additive prognostic role in the clinical outcomes in suspected VSA patients. Therefore, early risk stratification according to both ergonovine provocation test and NID would allow the identification of patients who may benefit from more aggressive means of treatment of vasospasm.

## Methods

### Study population

The suspected VSA patients who determined by clinicians to have suspicious symptoms (angina attacks at rest, particularly in the night and early morning or angina accompanied by ST-segment elevation intermittently or angina suppressed by CCB or nitrates) for VSA or clinical presentations (syncope or cardiac arrest or palpitation) possibly induced by VSA and, thus underwent coronary angiography (CAG) with the ergonovine provocation test were consecutively enrolled in this study. All potential study patients had normal findings or minimal (< 50% luminal diameter narrowing) atherosclerosis at the baseline CAG, whereas those with significant atherosclerosis (more than 50% luminal diameter narrowing) were excluded. Patients with renal failure (and treated with continuous dialysis), known malignant or inflammatory diseases, catheter-induced spasms, or significant fatal arrhythmias during baseline CAG were also excluded.

All patients with positive results on ergonovine provocation test received calcium channel blockers (CCBs) and/or other vasodilators (nitroglycerin) as part of the ongoing treatment. The research protocol conforms to the ethical guidelines of the 1975 Declaration of Helsinki and was approved by the Gil Medical Center Institutional Review Board (local IRB number: GIRBA2198). All patients provided written informed consent prior to enrollment.

### Intracoronary ergonovine provocation test

Vasodilator drugs such as CCBs and nitroglycerin were discontinued at least 48 h before CAG. After finishing baseline CAG on both coronary arteries, an intracoronary (IC) infusion of ergonovine was administered for the provocation test. For the left coronary artery provocation test, incremental ergonovine doses of 20 (E1), 40 (E2), and 60 µg (E3) were injected into the left coronary artery; alternatively, incremental ergonovine doses of 10 (E1), 20 (E2), and 40 µg (E3) were injected into the right coronary artery. If the patient’s condition was tolerable, left and right coronary artery provocation tests were both performed, sequentially. After the ergonovine provocation test, IC nitroglycerin (200 µg) was injected to evaluate NID.

### Quantitative coronary angiographic assessment

**Figure 6:**
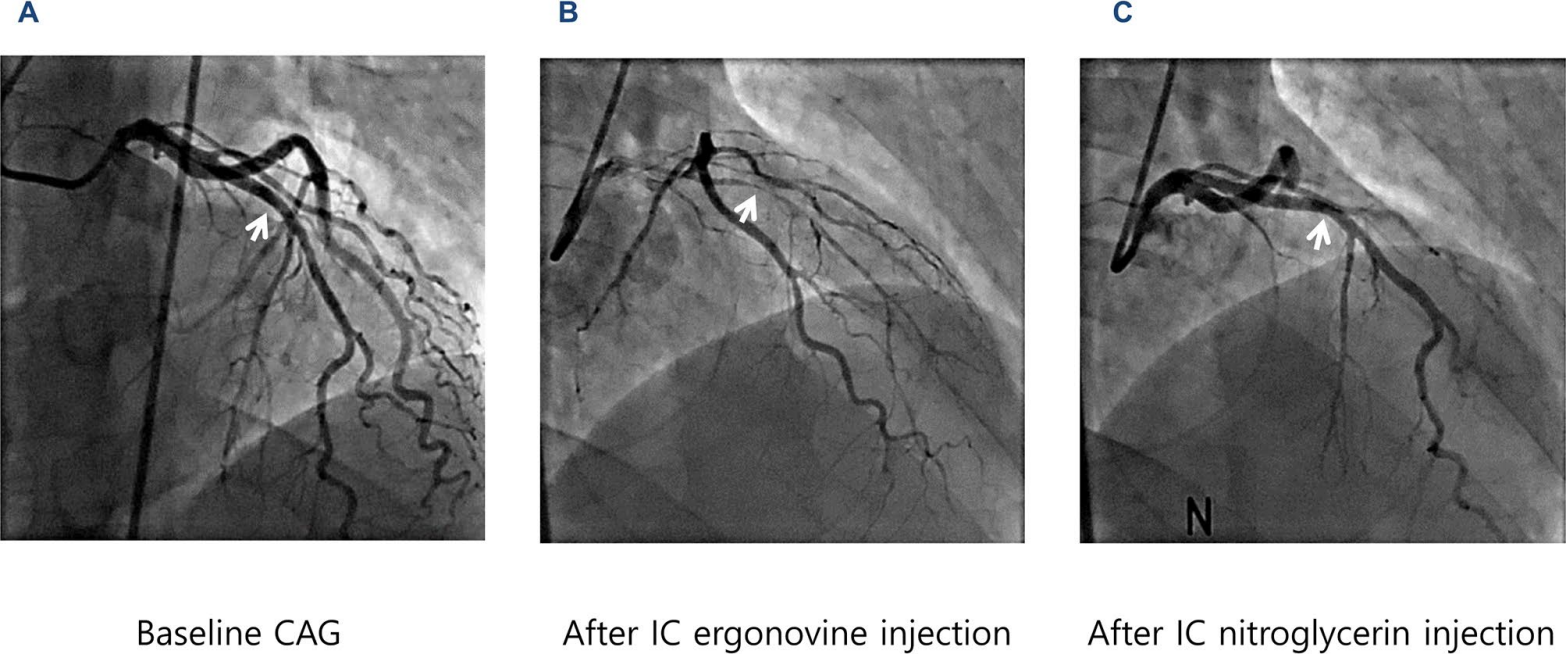
A representative case. (**A**) Baseline coronary angiography shows no significant stenosis in the left anterior coronary artery (LAD). (**B**) After IC ergonovine injection, significant narrowing occurs in the mid LAD. (**C**) After IC nitroglycerine injection, the previous significant narrowing in the mid LAD is resolved. The white arrow indicates the sites of maximal diameter change after IC ergonovine. Abbreviations: *CAG* coronary angiography, *IC* intracoronary, *LAD* left anterior descending coronary artery.

Diameters change after IC ergonovine injection and IC nitroglycerin injection were compared with baseline diameter at the same site of the coronary artery with the greatest diameter change after IC ergonovine injection (Fig. 6). Angiographic data were analyzed offline using a dedicated quantitative coronary angiography program (CAAS 5.9.2; Pie Medical Imaging B.V., Maastricht, The Netherlands) by two- independent investigators who were unaware of the patient's status.

### Definitions and study endpoint

Significant coronary artery spasm was defined as transient coronary artery occlusion (> 70% luminal diameter narrowing compared to baseline diameter) in response to an IC ergonovine stimulus, and the definition of VSA by ergonovine provocation test was significant coronary artery spasm with angina or ischemic ECG changes in response to IC ergonovine injection. An ischemic ECG change was defined as an ST-segment elevation or depression > 0.1 mV or a negative U-wave in at least two related leads.

We defined the positive result of NID as a more than 13.8% increase in luminal diameter after an IC injection of nitroglycerin compared with baseline coronary artery diameter, which was determined by receiver operating characteristic (ROC) curve analysis.

The primary endpoint of this study was defined as the major adverse cardiovascular event (MACE) comprising cardiac death, acute coronary syndrome (ACS), fatal arrhythmia, and syncope, and the secondary study endpoints were the individual components of the primary endpoint and re-hospitalization or emergency room visit for angina attack. Cardiac death was defined as any death due to cardiac causes such as myocardial infarction, low-output heart failure, fatal arrhythmia, and death from unknown causes. ACS was defined as recurrent or continuous chest pain lasting more than 20 min, with ischemic ECG change or elevation of cardiac markers including myocardial infarction. Fatal arrhythmia included symptomatic sustained ventricular tachycardia, ventricular fibrillation, and resuscitated cardiac arrest due to documented primary arrhythmia. All adverse events of interest were confirmed through the source documents, including medical records as well as telephone Interviews.

### Statistical analysis

Continuous data are expressed as mean ± standard deviation, and discrete variables are presented as absolute values and frequency (%). Continuous variables were compared using the two-sample *t*-test or ANOVA test and categorical variables using the chi-square test. Pearson's correlation coefficient (*r*) is used to assess the strength of the association between coronary diameter change after IC ergonovine and nitroglycerin. The ROC curve analysis of the NID result was performed on the result of a positive VSA result, which was determined by the ergonovine provocation test. The accuracy of the test was measured by the area under the ROC curve. The incidences of the primary endpoint in the positive and the negative groups of VSA and NID and its combinations were displayed with Kaplan–Meier curves and the Log-Rank test. Statistical significance was accepted for a two-sided value of *p* < 0.05. Statistical analyses were performed with SPSS version 20 (SPSS Inc., Chicago, IL, USA).

## Abbreviations

ACS: Acute coronary syndrome
CAG: Coronary angiography
CCBs: Calcium channel blockers
IC: Intracoronary
MACE: Major adverse cardiovascular event
NID: Nitroglycerin induced dilation
ROC: Receiver operating characteristic
VSA: Vasospastic angina
